# Evaluating the Clinical Relevance of Antibodies against Non-Human Leukocyte Antigen in Kidney Transplantation

**DOI:** 10.3390/antib13020044

**Published:** 2024-06-06

**Authors:** Shiv Bhutani, Shelley Harris, Michelle Carr, Marcus Russell-Lowe, Judith Worthington, Henry H. L. Wu, Rajkumar Chinnadurai, Kay Poulton

**Affiliations:** 1 Department of Renal Medicine, Manchester Royal Infirmary, Manchester University NHS Foundation Trust, Manchester M13 9WL, UK; 2 Department of Histocompatibility and Immunogenetics, Transplantation Laboratory, Manchester Royal Infirmary, Manchester University NHS Foundation Trust, Manchester M13 9WL, UK; shelly.harris@mft.nhs.uk (S.H.); michelle.carr@mft.nhs.uk (M.C.); marcus.russell-lowe@mft.nhs.uk (M.R.-L.); judith.worthington@mft.nhs.uk (J.W.); kay.poulton@mft.nhs.uk (K.P.); 3 Renal Research Laboratory, Kolling Institute of Medical Research, Royal North Shore Hospital & The University of Sydney, Sydney, NSW 2065, Australia; hon.wu@sydney.edu.au; 4 Department of Renal Medicine, Northern Care Alliance NHS Foundation Trust, Salford M6 8HD, UK; rajkumar.chinnadurai@nca.nhs.uk

**Keywords:** non-human leukocyte antibodies, graft rejection, kidney transplantation

## Abstract

*Introduction*: Kidney transplantation is the preferred modality of kidney replacement therapy for eligible patients with end-stage kidney disease (ESKD), given that it has been found to reduce mortality rates, improve quality of life, and is cost-effective compared to dialysis. Recent advancements in human leukocyte antigen (HLA) typing and donor-specific antibody (DSA) detection have helped to reduce the risk of rejection, but antibody-mediated rejection (AMR) can still occur without DSA. Previous studies suggest that rejection can be attributed to antibodies against Non-Human Leucocyte Antigens (non-HLAs). We aimed to acquire further understanding of the prevalence and distribution of non-HLA antibodies in our local population and attempt to correlate these findings with graft outcomes, as well as assess whether non-HLA antibodies can be utilized to determine graft impairment and dysfunction. *Methods*: We conducted a retrospective study involving kidney transplant recipients between January 2010 and December 2020. All included individuals were aged over 18 and underwent kidney-alone transplants; were ABO- and HLA-compatible; and were matched at A, B, and DR loci (mismatch 0:0:0). HLA testing was negative at the time of transplantation. The samples from both cases of early graft rejection and the control group were tested for non-HLA antibodies using One Lambda LABScreen^TM^, Autoantibody kit groups 1, 2, and 3, as well as the Immucor LIFECODES non-HLA autoantibody assay. *Results*: A total of 850 kidney transplant recipients were included, in which 12 patients experienced early graft rejection within the first month post transplant and 18 patients who did not experience graft rejection were selected as study controls. Our study reported no correlation between the total burden of non-HLA antibodies and early rejection, most likely as the result of a small sample size. Nevertheless, a sub-analysis revealed that specific high-frequency pre-transplant non-HLA antibodies such as GSTT, CXCL11, CXCL10, and HNR, detected by LIFECODES, were associated with rejection (Fisher’s exact test with Bonferroni correction, *p* < 0.001). Most pre-transplant non-HLA antibody levels were reduced after transplantation, which was attributed to immunosuppression. *Conclusion*: The ‘high frequency’ non-HLA antibodies displayed an association with graft rejection, though the overall associations between the burden of non-HLA antibodies and rejection episodes remain inconclusive. Further work is needed to establish the rebound phenomenon of non-HLA antibodies, the development of de novo non-HLA antibodies in the long run, and their implications on graft survival.

## 1. Introduction

Kidney transplantation is the definitive form of kidney replacement therapy for those living with end-stage kidney disease (ESKD) and associated with improved mortality and morbidity outcomes when compared to patients receiving dialysis [1]. Clinical outcomes following kidney transplantation may be affected by the presence of Donor-Specific Antibodies (DSAs) against the Human Leucocyte Antigens (HLAs) in the transplanted kidney. These antibodies can cause antibody-mediated rejection (AMR) in the transplanted kidney and shorten the kidney allograft survival [2]. However, in some patients, humoral rejection can still occur in the absence of DSA, and numerous studies have attributed the presence of graft rejection to the presence of antibodies against non-Human Leucocyte Antigens (non-HLAs) [3,4]. Whilst the role and relevance of HLA-specific antibodies in kidney transplantation and subsequent graft loss have been extensively studied, the frequency and clinical importance of non-HLA antibodies in kidney transplantation remain not fully understood [5,6]. Over the years, non-HLA antibodies have received sporadic attention with respect to their prevalence and clinical impact on the health trajectory of kidney transplant recipients. The causal factors of non-HLA antibody generation are very much unknown.

Under normal physiological conditions, antigenic determinants of targets for non-HLA antibodies are protected from immunological surveillance but become accessible after tissue injury or trauma. This injury could occur at pre-transplantation while on dialysis, pre-dialysis, during retrieval, or during allograft implantation or rejection. The release and presentation of non-HLA antigens at that time may induce an immune response. Non-HLA antibodies may be directed against a variety of antigens including minor histocompatibility antigens, vascular receptors, adhesion molecules, and intermediate filaments. For example, antibodies have been detected against Angiotensin II Type 1 receptor (AT1R), Endothelial-1 Type A receptors (ETARs), Polymorphic MHC class I-related chain A (MICA), and Vimentin antigens [7,8,9,10]. 

Kidney transplant recipients may carry non-HLA antibodies that could produce an immune response against cells in the microcirculation of the transplanted kidney, initiating an inflammatory process which may result in antibody-mediated allograft rejection [11,12]. The targets for these non-HLA antibodies are often cryptic and may also be subject to regional variation because of the genetic distribution across the local population and environmental factors [13]. The presence of non-HLA antibodies prompts a memory response and changes the active immune status of the patient, bringing about an overlap of alloimmunity and autoimmunity [14]. In this study, we aimed to gain a greater understanding on the prevalence and distribution of non-HLA antibodies in our local population and attempt to correlate these findings with graft outcomes, as well as assess whether non-HLA antibodies can be utilized as a clinical marker to determine graft impairment or dysfunction.

## 2. Materials and Methods

### 2.1. Study Design and Study Population 

We conducted a retrospective observational cohort study of adult patients (age > 18 years) who underwent kidney-only transplantation at the Manchester Royal Infirmary, United Kingdom, between 1 January 2010 and 31 December 2020. The Manchester Royal Infirmary is one of the largest kidney transplant hospital centers in the United Kingdom with an average of 275 kidney transplantation operations performed per year. 

**Figure 1:**
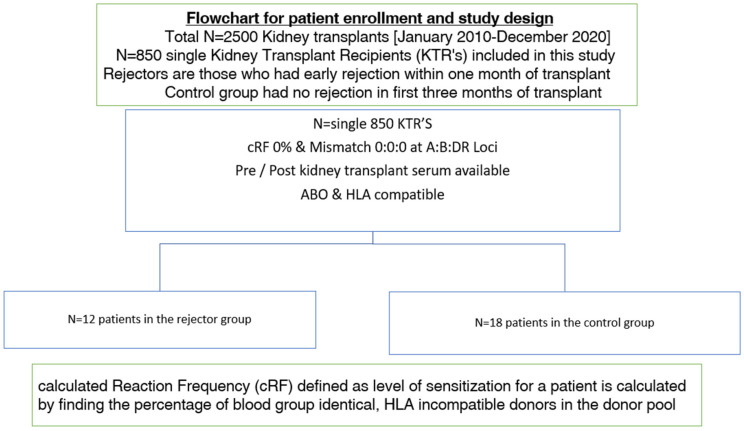
Flowchart describing study patient enrollment and study design.

From a total of around 2500 kidney transplant recipients over a 10-year period, 850 HLA-specific-antibody-negative kidney transplant recipients (all receiving their first kidney transplant) were assessed for this study. All transplant patients were ABO- and HLA-compatible; were matched at A, B, and DR loci (mismatch 0:0:0); and were HLA-negative at the time of transplant operation. Of these, patients experiencing early graft rejection (*n* = 12) were selected alongside a cohort of 18 patients who were randomly selected as controls (Figure 1). 

Kidney transplant recipients with early graft rejection were defined as those who had a kidney-biopsy-proven rejection within 1 month of kidney transplantation, and patients in the control group had no rejection within the first 3 months post transplant.

All patients had a pre-transplant serum sample drawn either on the day of transplant or within 90 days before the transplant, and a post-transplant serum sample drawn within 1 month after the transplant or at the time of kidney biopsy. 

The samples were investigated for non-HLA antibodies using the One Lambda, LABScreen^TM^ Autoantibody kit groups 1, 2, and 3 (LABScreen) [15,16], and the Immucor LIFECODES non-HLA autoantibody assay (LIFECODES) [17,18]. Both kits use Luminex bead technology and each kit covers a wide range of non-HLA antibody targets. The LABScreen kit covers 39 non-HLA antigen targets whilst the LIFECODES kit covers 60 non-HLA antigen targets.

The demographic parameters for each patient, such as age, gender, ethnicity, primary diagnosis, kidney replacement therapy (KRT) modality, dialysis vintage, type of transplant, rejection type (antibody, cellular, and mixed), and rejection severity, were collected prospectively and stored in our hospital’s electronic database. 

### 2.2. Detection of Non-HLA Antibodies 

The pre- and post-transplant serum samples were retrospectively tested for the presence of non-HLA antibodies using both LIFECODES and LABScreen autoantibody assays. For each non-HLA target, the cut-off MFI is defined by the vendor. Antibodies were assigned as positive for a given target if the calculated MFI was more than the pre-defined cut-off (or where MFI is >95%). 

### 2.3. One Lambda, LABScreen^TM^ Autoantibody Kit Groups 1, 2, and 3 Testing Technique

In relation to LABScreen autoantibody assay testing, raw data were collected using the LABScan™ 3D S066 instrument (LABScan 3D) (Thermo Fisher Scientific, Waltham, MA, United States. The sera were microfuged at 13,000 rpm for 10 min, and 20 µL of positive control, negative control, and test serum was added to the 5 µL autoantibody bead mix in a 96-well plate. The plate was covered with a tray seal and incubated for 30 min in the dark at room temperature. 

The plate was then washed with 150 μL of wash buffer followed by centrifugation at 1800× *g* for 5 min. The wash buffer was removed from the wells by flicking and the plate was washed twice more with 200 µL of wash buffer. An amount of 100 µL of 1× PE-conjugated goat antihuman IgG was added to each well (50 ng/µL) and mixed using a pipette. The tray was covered with a tray seal and incubated for 30 min in the dark at room temperature. The plate was then washed three times as before using 200 µL of wash buffer.

Eighty microliters of PBS was added to each well and mixed using a multichannel pipette. The beads were then transferred to a low-profile plate and transferred to the LABScan™ 3D. Data acquisition was performed using FLEXMAP 3D^®^ software version 4.2 and the LABScreen results were analyzed using the HLA Fusion Research software V6.4.

### 2.4. Immucor LIFECODES Non-HLA Autoantibody Assay Testing Technique 

For Immucor LIFECODES non-HLA autoantibody assay testing, the sera were microfuged at 13,000 rpm for 10 min, and 10 μL of positive control, negative control, and test serum was added to 40 μL of LIFECODES beads in a 96-well plate and mixed using a pipette. The plate was then covered with a tray seal and incubated for 30 min in the dark at room temperature. The plate was then washed with 150 μL of wash buffer followed by centrifugation at 1300× *g* for 5 min. The wash buffer was removed from the wells by flicking and the plate was washed twice more with 200 µL of wash buffer.

The conjugate as provided with the kit was diluted 1:10 in wash buffer. Fifty microliters of conjugate was added to each well and mixed. The plate was covered with a tray seal and incubated at room temperature in the dark for 30 min. This plate was then washed twice with 200 μL of wash buffer.

One hundred and fifty microliters of wash buffer was added to the wells. The beads were resuspended by mixing with a pipette and then transferred to a low-profile 96-well plate. Data acquisition was performed using the LABScan™ 3D with FLEXMAP 3D^®^ software and the results were analyzed using the LIFECODES non-HLA antibody analysis tool.

### 2.5. Kidney Allograft Biopsies, Histological Grading, and Treatment of Graft Rejection

In this cohort, we included all kidney transplant recipients where there was an indication for allograft biopsy during the post-transplant follow-up within 1 month of kidney transplantation. The biopsies were performed at the time of graft dysfunction (i.e., occurrence of eGFR decline, proteinuria, or delayed graft function). All biopsies were reviewed and scored for T-cell-mediated rejection (TCMR) and antibody-mediated rejection (AMR) by an accredited pathologist according to the most recent Banff 2019 consensus [19]. 

### 2.6. Statistical Analysis 

Descriptive statistics are reported using numbers and percentages for categorical variables and the Chi-square test was used to determine *p*-values. The continuous variables are expressed as the median and interquartile range and the Mann–Whitney U test was used to determine *p*-values. Univariable and multivariable logistic regression analysis was used to estimate the relationships between different patient factors and the presence of pre- and post-transplant non-HLA antibodies. A *p*-value of less than 0.05 was considered statistically significant. The Fisher’s exact test was used to determine whether there were non-random associations between two categorical variables. The Bonferroni correction was also used for adjustment for multiple tests to calculate the adjusted *p*-value. Data were analyzed using IBM SPSS-version 26, registered with the University of Manchester.

## 3. Results

### 3.1. Demographic Data of the Study Population 

Of the 850 kidney transplant recipients who underwent screening, 30 patients were selected for further analyses for the purposes of this study. There were 12 patients who had early graft rejection (i.e., rejection occurred <1 month following transplantation) and 18 patients were controls as per the study inclusion criteria. A total of 23 patients (77%) received a deceased donor kidney allograft whilst the other 7 patients (23%) received a living donor transplant. 

The mean age of the cohort was 52 years with a predominance of males (70%) and individuals of white ethnicity (77%). The median dialysis vintage of the cohort was 26 months. Overall, no statistically significant differences were observed between the baseline characteristics in the two groups (Table 1). 

In total, 60 serum samples (30 pre-transplant; 30 post-transplant) of the selected cohort were available and tested for the presence of different non-HLA antibodies using LABScreen and LIFECODES autoantibody assays. 

### 3.2. Pre and Post-Transplant Non-HLA Antibodies in Early Graft Rejection and Control Groups Using LABScreen Autoantibody Assay Testing Kit

**Figure 2:**
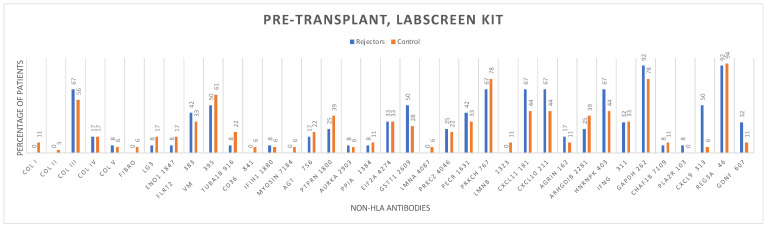
Pre-transplantation non-HLA antibodies in the early rejectors and control groups using the LABScreen autoantibody assay testing kit.

**Figure 3:**
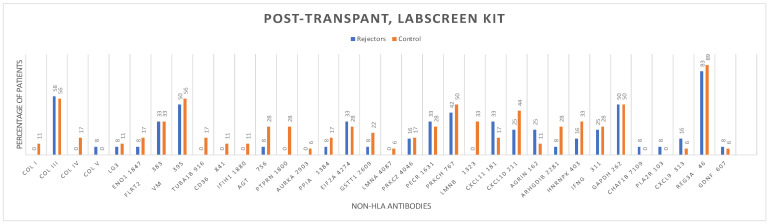
Post-transplantation non-HLA antibodies in the early rejectors and control groups using the LABScreen autoantibody assay testing kit.

The distribution of non-HLA antibodies in the graft rejection and control groups before and after kidney transplantation, using the LABScreen One Lambda Kit from Canoga Park, CA, USA, is shown (Figure 2 and Figure 3). 

Logistic regression analysis found no statistically significant association between the pre-transplant (HR = 1.03; 95%CI 0.89−1.17; *p* = 0.69) and post-transplant non-HLA antibodies (HR = 0.89; 95%CI 0.73–1.08; *p* = 0.25) tested by the LABScreen autoantibody assay testing kit and biopsy-proven rejection (Table 2).

### 3.3. Pre and Post-Transplant Non-HLA Antibodies in Early Graft Rejection and Control Groups Using LIFECODES Autoantibody Assay Testing Kit

**Figure 4:**
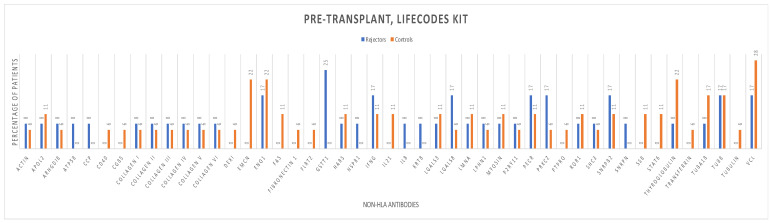
Pre-transplantation non-HLA antibodies in the early rejectors and control groups using the LIFECODES autoantibody assay testing kit.

**Figure 5:**
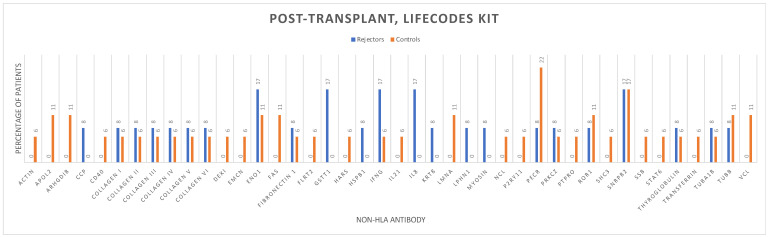
Post-transplantation non-HLA antibodies in the early rejectors and control groups using the LIFECODES autoantibody assay testing kit.

The distribution of pre- and post-transplant non-HLA antibodies for patients in both the early graft rejection and control groups is shown (Figure 4 and Figure 5), which represents the broadness of non-HLA immunity before and after kidney transplantation. 

Logistic regression analysis (Table 2) demonstrated no statistically significant association between pre-transplant (HR = 1.01; 95%CI 0.83–1.23; *p* = 0.91) and post-transplant non-HLA antibodies (HR = 0.95; 95%CI 0.73–1.25; *p* = 0.75) tested by the LIFECODES testing kit and biopsy-proven rejection.

### 3.4. Association between High-Frequency Non-HLA Antibodies and Graft Rejection 

The LABScreen and LIFECODES testing kits use varying MFI cut-offs for each non-HLA antibody, as defined by the vendor. The antibodies are classified as ‘high frequency’ non-HLA antibodies based on their occurrence in the early graft rejection and control groups.

For non-HLA antibodies evaluated with the LABScreen testing kits, antibodies which are found in at least 50% of the early graft rejection group are classified as ‘high frequency’. A two-tailed Fisher’s exact test concluded that there is a significant association between graft rejection and certain antibodies, including GSTT 2609, CXCL11, CXCL10, HNRN PK, and CXCL9 (Table 3). As 10 different antibodies were investigated, the threshold for significance was <0.005 after Bonferroni correction. As per the adjusted *p*-value, GSTT1, CXCL11, and CXCL10 demonstrated significant associations with graft rejection. 

For non-HLA antibodies tested with the LIFECODES testing kit, ‘high frequency’ non-HLA antibodies are defined as those with a prevalence of 15% or higher in the graft rejection group. A two-tailed Fisher’s exact test concluded that there is no significant association between ‘high frequency’ non-HLA antibodies and graft rejection (Table 4). 

### 3.5. Trajectory in Non-HLA Antibody Levels in Pre- and Post-Transplant States

Our center follows a standard immunosuppression regime for kidney transplant recipients which includes a prescription of Basiliximab on day 0 and day 4 following the transplant operation as an induction agent, and then maintenance therapy with Tacrolimus, Mycophenolic acid, and +/− Prednisolone based on the patient’s body weight and immunological risk. Autoantibody assay testing using the LABScreen and LIFECODES testing kits both found a reduction in total antibody load for the early graft rejection and control groups after kidney transplantation (Table 5 and Table 6). 

### 3.6. Other Predictors of Graft Rejection 

Our study explored other potential predictors for graft rejection alongside the relevance of non-HLA antibodies within this context. It is noted that age, gender, ethnicity, and type of KRT were not linked to early graft rejection (cellular, humoral, or mixed) within the first month after kidney transplantation (Table 2). 

## 4. Discussion

Over a 10-year period between January 2010 and December 2020, our center successfully performed 850 cases of single kidney transplantation that were unsensitized (i.e., calculated reaction frequency (0%) and completely matched at A:B: DR loci (0:0:0)). We identified cases of early graft rejection and study controls based on predetermined criteria, and we examined them for their pre- and post-kidney transplant non-HLA antibody response to a wide range of non-HLA antigens using two different autoantibody assay testing kits—LABScreen and LIFECODES. 

Whilst the presence of non-HLA antibodies may have increased the risk of donor DSA and subsequently graft rejection, our study did not detect any significant correlation between the total burden of non-HLA antibodies and early graft rejection, which was probably explained by a small study sample size [3,4]. Nevertheless, a sub-analysis revealed that specific ‘high frequency’ pre-transplant non-HLA antibodies including GSTT1, CXCL11, CXCL10, and hnRNPK detected by LIFECODES testing were associated with graft rejection. Given the variability in non-HLA antibody targets and tissue expression patterns, there is no specific histopathological feature or phenotype alone, or when combined with HLA-DSA, that is specific towards non-HLA antibodies and their expression patterns [20]. 

Few non-HLA antibodies have been extensively studied in the medical literature, but its implications on graft survival and patient morbidity have been observed in solid organ transplantation (SOT) and hemopoietic stem cell transplantation (HSCT). Aguillera et al. [21] reported a study involving 419 liver transplant recipients. Seventy percent of patients who had a mismatch between the Glutathione S-transferase theta 1 (GSTT1) genotype and their donor developed de novo immune-mediated hepatitis and transplant dysfunction. Similarly, Comoli et al. [22] observed harmful effects of GSTT1Abs—alone or in combination with HLA-DSAs—on kidney graft outcome. Yeo et al. [23] explored the role of GSTT1 in cardiothoracic transplantation and demonstrated that pre-transplant anti-GSTT1 antibodies that developed post transfusion are associated with a higher incidence of acute allograft rejection. Martinez-Bravo et al. [24] investigated the production of anti-GSTT1 antibodies and their involvement in hepatic Graft-versus-Host Disease (GvHD) post HSCT. Furthermore, GSTT1 donor/recipient mismatches are associated with an increased risk of developing acute and chronic GvHD.

Also, the role of anti-heterogeneous nuclear ribonucleoprotein-K (hnRNPK) antibodies was studied in solid organ transplants. A study by Acevedo et al. [25] explored the significance of anti-hnRNPK antibodies, a type of non-HLA antibody, in the development of cardiac allograft vasculopathy (CAV) in heart transplant recipients. CAV is diagnosed through angiography and intravascular ultrasound (IVUS) technology. This study analyzed sera samples from 48 heart transplant recipients in detecting the presence of antibodies. The investigators identified hnRNPK as a new antigenic target for developing CAV. Anti-hnRNPK antibodies were found to be significantly higher in patients diagnosed with CAV through IVUS or angiography compared to patients without CAV. This study suggests that anti-hnRNPK antibodies are statistically associated with CAV disease following heart transplantation, regardless of the diagnostic technique applied.

During kidney transplantation, a sudden increase in immune cells entering the allograft would be expected. This process is facilitated by the upregulation of endothelial adhesion molecules and the release of chemokines from parenchymal cells. Krupicekova et al. [26] found that 44 patients with acute allograft rejection had higher serum levels of chemokines (i.e., CXCL1, CXCL5, CXCL6, CCL2, CCL21, and particularly CXCL10; and CX3CL1 before transplantation), which suggested their higher inflammatory state. Samples collected on the day of kidney biopsy displayed positive results for acute rejection. The chemokines CXCL9 and CXCL11 were found to be upregulated, which would attract Type 1 T-helper lymphocytes. Additionally, patients with acute rejection had significantly higher CXCL10 concentrations at 1-month and 1-year follow-ups post transplant, in comparison to patients with no post-transplant clinical complications.

Ultimately, our study cohort is relatively small to draw any significant correlation between the presence of non-HLA antibodies and histopathological changes in graft rejection. Although our results do highlight the potential relevance of serum reactivity to a single non-HLA autoantigen, more work is needed to determine the exact significance and pathogenic potential of these findings, and whether there are any practice-changing implications. 

Throughout this study, we applied a standard induction and maintenance immunosuppression protocol for kidney transplant recipients in our center, and the cross-sectional analysis revealed that the majority of pre-transplant non-HLA antibody levels were reduced following transplantation. This could have been attributed to the approach of induction and maintenance immunosuppression here. Further longitudinal data to increase the understanding of the rebound phenomenon of non-HLA antibodies, the development of de novo non-HLA antibodies following kidney transplantation, and their implications on graft survival are required. As the required kits to stain for non-HLA antibodies in our graft rejection biopsy samples were not available, direct evidence of the involvement of non-HLA antibodies in graft rejection could not be provided. 

We used testing kits from two manufacturers to test for non-HLA antibodies for this study. However, from our small cohort sample size, it was difficult to draw clearly defined interpretations of the impact of this measure. The lack of concordance between the two kit test procedures could be due to the different detection sensitivity, range, or epitope coverage amongst other factors. The kit for LIFECODES testing appears to be more sensitive in identifying rejection-associated antibodies. Going forward, longer-term basic and translational studies are necessary to determine the associations between non-HLA antibodies and acute tissue injury and to distinguish the effects of persistent and de novo non-HLA antibodies on graft survival. The introduction of follow-up confirmatory assays to validate the ‘high frequency’ non-HLA antibodies via other orthogonal quantitative methods would be ideal. Ultimately, the emerging availability of commercial kits to test for non-HLA antibodies in a transplant setting is very encouraging from a translational perspective. It will increasingly help transplant programs evaluate non-HLA antibodies and HLA-DSA to stratify post-transplant immunological risks. The clinical validation of these kits with a standardized quality control approach in larger populations is anticipated [20]. 

## 5. Conclusions 

In summary, non-HLA antibodies are less polymorphic and not as widespread as HLA antibodies. Our study identified numerous ‘high frequency’ non-HLA antibodies that displayed an association with graft rejection, though the overall associations between the burden of non-HLA antibodies and rejection episodes remain inconclusive. Prospective multi-center studies with larger sample sizes are needed to clarify further the role and relevance of non-HLA antibodies in kidney transplantation. 

## Figures and Tables

**Table 1:** Baseline characteristics and follow-up of the study population.

Variable	Total (*n* = 30)	Rejectors (*n* = 12)	Control (*n* = 18)	*p*-Value
Age, years	52 (41–60)	46 (33–62)	54 (43–60)	0.39
Gender, Male	21 (70%)	8 (66.7%)	13 (72.2%)	0.74
Ethnicity, White	23 (76.7%)	9 (75%)	14 (77.8%)	0.86
*Primary diagnosis*				0.89
Hypertension	6 (20%)	2 (16.7%)	4 (22.2%)	
Diabetes Mellitus	6 (20%)	3 (25%)	3 (16.7%)	
Cystic kidney disease	4 (13.3%)	2 (16.7%)	2 (11.1%)	
GN/Vasculitis	10 (33.3%)	3 (25%)	7 (38.9%)	
Other	4 (13.3%)	2 (16.7%)	2 (11.1%)	
*KRT status pre-transplant*				0.42
Pre dialysis	10 (33.3%)	3 (25%)	7 (38.9%)	
Dialysis	20 (66.7%)	9 (75%)	11 (61.1%)	
Dialysis Vintage, months	26 (13.5–41.5)	22.2 (13.2–40.5)	26.5 (18–39)	0.75
*Type of transplant*				0.53
DBD	11 (36.7%)	3 (25%)	8 (44.4%)	
DCD	12 (40%)	6 (50%)	6 (33.3%)	
LD	7 (23.3%)	3 (25%)	4 (22.2%)	

DBD: Donation after brainstem death; DCD: Donation after cardiac death; GN: Glomerulonephritis; KRT: kidney replacement therapy; LD: Live donor.

**Table 2:** Univariate logistic regression analysis to evaluate the predictors of graft rejection.

Variable	OR (95% CI)	*p*-Value
Age, years	0.97 (0.91–1.02)	0.26
Gender, Male	0.77 (0.16–3.7)	0.74
Ethnicity, White	0.86 (0.15–4.7)	0.86
RRT status per-Transplant, Dialysis	1.91 (0.38–9.5)	0.43
LABScreen pre-transplant, Number of antibodies	1.03 (0.89–1.17)	0.69
LABScreen post-transplant, Number of antibodies	0.89 (0.73–1.08)	0.25
LIFECODES pre-transplant, Number of antibodies	1.01 (0.83–1.23)	0.91
LIFECODES post-transplant, Number of antibodies	0.95 (0.73–1.25)	0.75

LABScreen: One Lambda, LABScreen^TM^ Autoantibody kit groups 1, 2, and 3; LIFECODES: Immucor LIFECODES non-HLA autoantibody assay.

**Table 3:** Association between high-frequency pre-transplant non-HLA antibodies and graft rejection determined by LABScreen autoantibody assay testing.

Rejectors	*n*	Controls	*n*	*p*-Value	Adjusted *p*-Value with Bonferroni Correction (<0.005 Is Significant)
COL III	8	COL III	10	0.71	
VM	6	VM	11	0.71	
GSTT1	6	GSTT1	0	0.0016	Significant
PRKCH	8	PRKCH	14	0.68	
CXCL11	8	CXCL11	0	0.0001	Significant
CXCL10	8	CXCL10	0	0.0001	Significant
hnRNPK	8	hnRNPK	0	0.0016	Significant
GAPDH	11	GAPDH	14	0.62	
CXCL9	6	CXCL9	0	0.002	
REG3A	11	REG3A	17	1.000	

**Table 4:** Association between high-frequency pre-transplant non-HLA antibodies and graft rejection determined by LIFECODES autoantibody assay testing.

Rejectors	*n*	Controls	*n*	*p*-Value
ENO1	2	ENO1	0	1.000
GSTT	3	GSTT	0	0.054
IFNG	2	IFNG	0	0.15
LGALS8	2	LGALS8	0	0.15
PECR	2	PECR	0	0.15
PRKCZ	2	PRKCZ	0	0.15
SNRPB2	2	SNRPB2	0	0.15
TUBB	2	TUBB	3	1.000
VCL	2	VCL	3	1.000

**Table 5:** Trajectory of non-HLA antibody load after kidney transplantation determined by LABScreen autoantibody assay testing in pre- and post-transplant samples.

	Rejectors (n%)	Control (n%)	Total Antibodies (n%)
Increased load	8%	28%	20%
Reduced load	92%	50%	67%
Same load	0%	22%	13%

**Table 6:** Trajectory of non-HLA antibody load after kidney transplantation determined by LIFECODES autoantibody assay testing in pre- and post-transplant samples.

	Rejectors (n%)	Control (n%)	Total Antibodies (n%)
Increased load	8%	11%	10%
Reduced load	58%	56%	57%
Same load	33%	33%	33%

## Data Availability

Data are contained within the article.
